# Diverse Intercropping Patterns Enhance the Productivity and Volatile Oil Yield of *Atractylodes lancea* (Thunb.) DC.

**DOI:** 10.3389/fpls.2021.663730

**Published:** 2021-07-20

**Authors:** Zheng Peng, Yan Zhang, Binbin Yan, Zhilai Zhan, Xiulian Chi, Yang Xu, Xiuzhi Guo, Xinping Cui, Tielin Wang, Sheng Wang, Chuanzhi Kang, Xiufu Wan, Kai Sun, Luqi Huang, Lanping Guo

**Affiliations:** ^1^State Key Laboratory of Dao-di Herbs, National Resource Center for Chinese Materia Medica, China Academy of Chinese Medical Sciences, Beijing, China; ^2^Institute of Healthcare China Academy of Chinese Medical Sciences, Nanchang, China

**Keywords:** *Atractylodes lancea* (Thunb.) DC., intercropping, survival, production, volatile oil

## Abstract

Commercial cultivation of the medicinal plant *Atractylodes lancea* is significantly restricted by low survival rates and reduced yields. Intercropping can reasonably coordinate interspecific interactions, effectively utilize environmental resources, and increase survival and yield. We conducted a field experiment from 2014 to 2016 to analyze the advantages and effects of intercropping on *A. lancea* survival, growth traits, individual volatile oil content, and total volatile oil content. In addition to *A. lancea* monoculture (AL), five intercropping combinations were planted: *Zea mays* L. (ZM) + *A. lancea*, *Tagetes erecta* L. (TE) + *A. lancea*, *Calendula officinalis* L. (CO) + *A. lancea*, *Glycine max* (Linn.) Merr. (GM) + *A. lancea*, and *Polygonum hydropiper* L. (PH) + *A. lancea*. The survival and average rhizome weight of *A. lancea* was higher in the ZM, CO, and TE treatments than in the monoculture treatment, and the average plant height was higher in all intercropping treatments than in the monoculture. The volatile oil content of *A. lancea* from the ZM and CO treatments was significantly improved relative to that of monoculture plants. The volatile oil harvest was higher in the ZM, CO, and TE treatments than in the monoculture. We conclude that intercropping is an effective way to increase the survival and yield of *A. lancea*. Furthermore, intercropping with ZM, CO, and TE increases the harvest of four volatile oils from *A. lancea*.

## Introduction

The rhizomes of *Atractylodes lancea* (Thunb.) DC. (Chinese: Cangzhu) are commonly used in traditional Chinese medicine as a remedy for rheumatic diseases, digestive disorders, night blindness, and influenza (Oseko et al., 2005). In the past few decades, the demand for *A. lancea* has been increasing, as the use of its active compounds in the pharmaceutical industry has grown substantially. During the 2020 novel coronavirus pneumonia outbreak, *A. lancea* was one of the main traditional Chinese medicinal materials used for the prevention of COVID-19 infection (Yang et al., 2020; Zhao et al., 2020). *A. lancea* is a perennial plant that is typically harvested from the field after 2 – 3 years of cultivation. Because wild *A. lancea* resources are increasingly endangered, the market depends heavily on artificial cultivation. However, industrial *A. lancea* monoculture systems face great hazards associated with continuous cropping, including the suppression of soil fertility, reduced productivity, and increased pest and disease damage. The disease incidence rate on *A. lancea*, especially that of root rot disease, can reach up to 80%, causing serious reductions in growth and productivity (Wang et al., 2016). Guo et al. (2005) have previously reported that autotoxicity may be another negative effect of the continuous cropping of single cultivars.

Compared with the planting of single cultivars, intercropping has significant agro-ecological advantages (Power, 1989; Khan et al., 1997; Brooker et al., 2015). The disease problems associated with continuously cropping patchouli can be ameliorated by intercropping with turmeric and ginger (Zeng et al., 2020). Maize/soybean intercropping suppressed the occurrence of soybean red crown rot (Gao et al., 2014), and maize/pepper intercropping can reduce disease levels of soil-borne *Phytophthora* on pepper (Yang et al., 2015). Intercropping Chinese chive cultivars with banana can reduce the incidence of Panama disease (Li Z. et al., 2020). Traditional intercropping usually aims at the improvement of crop yields (Li C. et al., 2020), which consist mainly of primary metabolites. By contrast, the aim of medicinal plant cultivation is usually the production of more secondary metabolites. Previous research has reported that intercropping may lead to changes in plant accumulation of secondary metabolites (Maffei and Mucciarelli, 2003; Ngwene et al., 2017; Zeng et al., 2020).

In this study, we carried out two years of field experimentation to determine the effects of intercropping on *A. lancea*. The objectives of the study were: (i) to compare important growth indicators in different intercropping systems; (ii) to compare plant yield and the accumulation of secondary metabolites in different intercropping systems; and (iii) to investigate the different accumulation patterns of major active components under five intercropping systems.

## Materials and Methods

### Experimental Site

Field experiments were conducted on newly developed terraces in Huadun village, Laibang Town, Yuexi County, Anhui Province (30°56′7.15′′N, 116°1′40.43′E, altitude 620 m) in 2015 and 2016. This site is located in the north subtropical humid monsoon climate area, and its frost-free period is 220 days. The mean annual temperature is 17°C, the mean annual ground temperature is 17°C, the average annual precipitation is 2434.6 mm, and the average sunshine duration is 2070.5 h. Meteorological data from 2015 and 2016 were collected by automatic weather stations near the test site.

### Experiment Design and Field Management

In this study, intercropping partners were selected on the basis of their functions. Selected species are all common native and agricultural species. The gramineous roots of *Zea mays* L. (ZM) can activate soil microbial flora (Abbott and Robson, 1991), and its aboveground parts are tall and dense, providing a degree of shade. Both marigold and calendula are from the *Asteraceae* family, and their above- and belowground parts contain volatile oils that provide resistance to pests and diseases (Mansoor and Mashkoor, 1988; Natarajan et al., 2006). It has been shown that *Calendula officinalis* L. (CO) can be used for pest control, and the calendula oil contained therein can be used as a repellent to prevent egg laying by flies (Pudasaini et al., 2008; Riaz et al., 2009). Extracts of *Tagetes erecta* L. (TE), leaves, and roots are toxic to the nematode that is closely associated with root rot (Wang et al., 2003; Hooks et al., 2010). The *Glycine max* (Linn.) Merr. (GM) root system harbors nitrogen-fixing rhizobia and has the effect of enhancing soil fertility. Finally, *Polygonum hydropiper* L. (PH) is the natural companion species of *A. lancea*.

Seedlings of *A. lancea* were derived from *A. lancea* rhizomes growing in Huoshan, Anhui Province, and seedlings of similar size were used for the experiment. Seeds of maize, soybean, marigold, calendula, and *P. hydropiper* were those of commercial cultivars.

Five intercropping treatments and an *A. lancea* monoculture treatment were used in the experiment: *A. lancea* alone, *A. lancea* + *Zea mays* L. (ZM), *A. lancea* + *Glycine max* (Linn.) Merr. (GM), *A. lancea* + *Tagetes erecta* L. (TE), *A. lancea* + *Calendula officinalis* L. (CO), and *A. lancea* + *Polygonum hydropiper* L. (PH). The row spacing between *A. lancea* and *A. lancea* is 20 × 30 cm, and the row spacing between *A. lancea* and partner plants is 20 × 30 cm, and the spacing between partner plants and partner plants is also 20 × 30 cm. The experiment used a randomized complete block design with four replications, and each experimental plot was 10 m^2^ (2 m × 5 m). Therefore, there are 187 *A. lancea* in AL treatment and 99 *A. lancea* in intercropping treatment.

All plants were planted by hand. *A. lancea* was only planted in December 2014 and has been grown in the field for two years. And the partner plants were planted for the first time in April 2015 and the second planted in April 2016. *A. lancea* was planted by rhizome propagation after sterilization (soaking for 30 min at room temperature in 50% carbendazim diluted 800–1000 times), and rhizomes were buried 1–2 cm underground. Intercropping plants were grown from seed sown 1–2 cm deep. The plantings were weeded in March, June, and November of each year, and no pesticides or fertilizers were used throughout the experimental period.

### Measurement Parameters and Methods

#### Plant Biomass and Yield

At the end of November 2015 and 2016, ten *A. lancea* were selected from each experimental plot for biomass and yield analysis, including both their above- and belowground parts. Measurements included plant height, number of branches, number of apical and lateral buds on the rhizome, and rhizome fresh weight.

#### Collection and Analysis of Volatiles

*Atractylodes lancea* rhizomes were collected and dried in a 40°C oven for one week to constant weight, then crushed to <0.3 mm. A 500-mg sample of the resulting powder was placed into a 50-ml centrifuge tube, 25 ml of n-hexane was added, and the mixture was first shaken (250 min^–1^, 15 min) and then centrifuged at 3000 rpm for 10 min. The supernatant was removed for subsequent use, 20 ml of n-hexane was added, and the above process was repeated. Both supernatants were combined in a volumetric flask, 1.0 ml of the internal standard solution was added, the sample was diluted to 50 ml, and a 1-ml sample was injected into the gas chromatograph/mass spectrometer (GC/MS) (Trace 1310 gas chromatograph and TSQ 8000 mass spectrometer).

**FIGURE 1:**
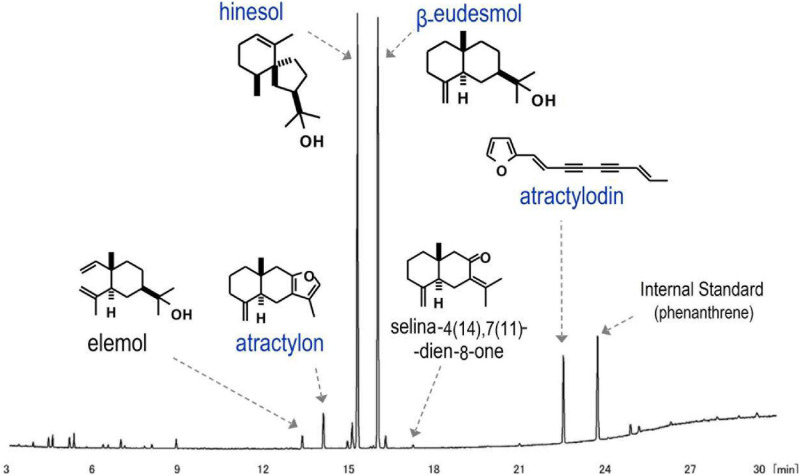
Typical GC-MS chromatogram of several classical volatile oils presents in *A. lancea*.

The contents of four volatile active ingredients, atractylon, hinesol, β-camphor (β-eudesmol), and atractylodin, were measured using the method of Li (2018) with an Agilent DB-5ms series column (0.25 mm id × 30 m, 0.25 μm). The carrier gas was helium (flow rate: 1 ml/min), the injection mode was split (proportion 50:1), the injection volume was 1 ml, and the inlet temperature was 240°C. The column temperature was 120°C for the first 2 min; then the temperature was programmed to rise to 240°C at 5°C/min and was maintained at 240°C for 5 min. The MSD ionization mode had the following parameters: ionization voltage (EI) 70 V, ion source 230°C, and quadrupole 150°C. The MSD data acquisition mode was scanning (40–500 AMU). As shown in Figure 1, the method is accurate, fast, and reproducible.

### Data Analysis

Tukey’s HSD test was used to test for differences among intercropping treatments. Pearson’s correlation coefficient was used to analyze the correlations among *A. lancea* growth and biochemical indices under different intercropping treatments. Statistics and correlation analysis were performed using SPSS v16.0 (SPSS Inc., Chicago, United States) and Microsoft Excel 2003.

## Results

### Effects of Different Intercropping Treatments on the Survival of *A. lancea*

**FIGURE 2:**
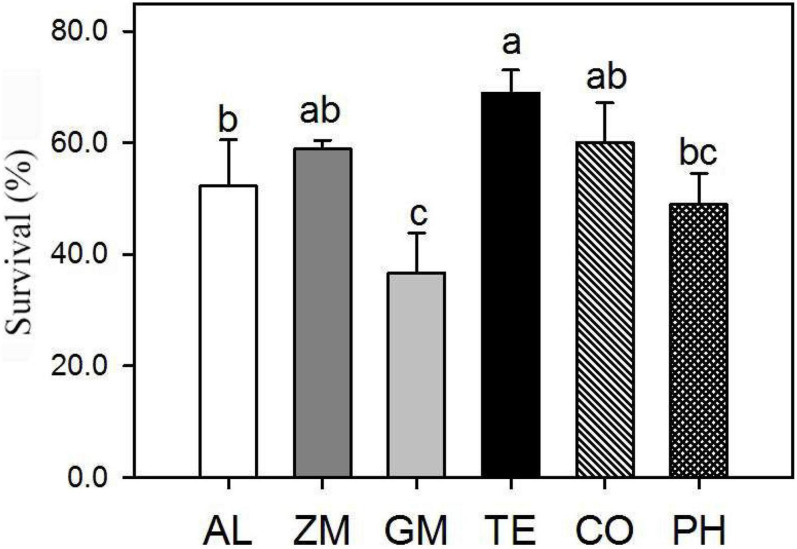
Effect of different intercropping treatments on the percentage survival of *A. lancea* in 2016. Different lowercase letters indicate significant differences at the 5% significance level.

The survival percentage of *A. lancea* from different treatments was investigated two years after planting (Figure 2). Plant survival was significantly higher in the TE treatment (69 ± 4.2%) than in the monoculture (52 ± 8.3%), followed by the CO (60 ± 7.2%) and ZM (59 ± 1.6%) treatments. By contrast, the survival of plants in the PH (49 ± 5.7%) and GM (37 ± 1.8%) treatments was significantly lower than that of monoculture plants.

### Effects of Different Intercropping Treatments on Growth and Yield of *A. lancea*

**FIGURE 3:**
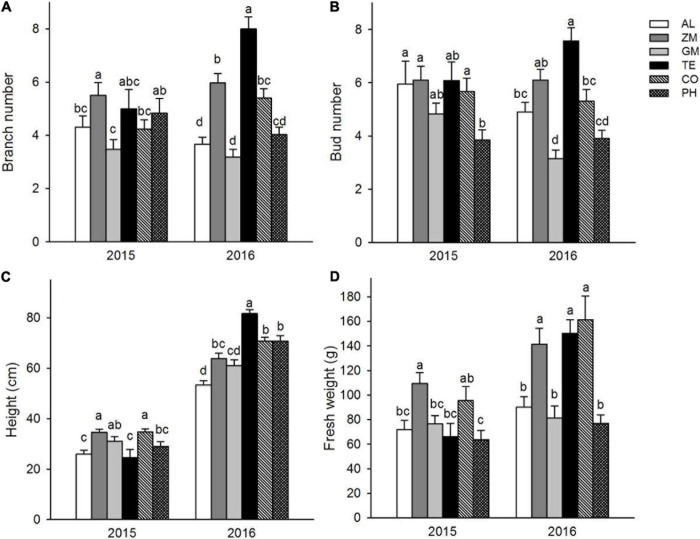
Effect of different intercropping treatments on branch number **(A)**, bud number **(B)**, height **(C)**, and fresh weight **(D)** of *A. lancea* in 2015 and 2016. Different lowercase letters indicate significant differences at the 5% significance level.

In 2015, the average plant height was significantly greater in the ZM and TE treatments (both ∼34 cm) than in the monoculture (26 ± 1.6 cm) (Figure 3). Likewise, the average rhizome weight was significantly greater in the ZM and TE treatments (109 ± 8.8 g and 96 ± 11.3 g, respectively) than in the monoculture (72 ± 7.5 g) (Figure 3). In 2016, the average plant height was significantly greater in all the intercropping treatments than in the monoculture (Figure 3). Similarly, the average rhizome weights in the ZM, TE, and CO treatments were 141 ± 13.0 g, 150 ± 10.9 g, and 161 ± 19.2 g, all significantly greater than that in the monoculture (90 ± 8.7 g) (Figure 3).

### Effects of Different Intercropping Treatments on the Concentrations of Volatile Oils in *A. lancea*

**FIGURE 4:**
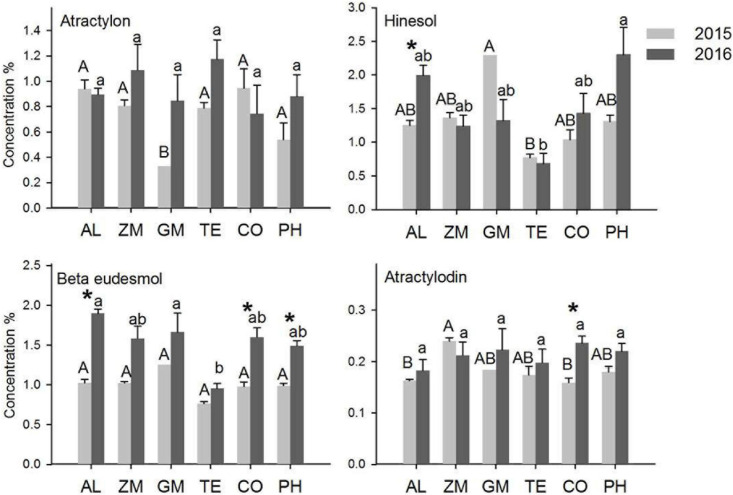
The effect of different intercropping treatments on the concentrations of four volatile oils from *A. lancea* in 2015 and 2016. Different capital (2015) and lowercase (2016) letters indicate significant differences at the 5% significance level, and asterisks indicate significant differences between 2015 and 2016 within individual treatments.

The concentrations of the four main volatile oils in *A. lancea* were analyzed in 2015 and 2016 (Figure 4). The concentration is the ratio of the mass of volatile oil to the mass of *A. lancea* rhizome. In 2015, the atractylon concentration of *A. lancea* was lowest in the GM treatment, the hinesol concentration of *A. lancea* was lowest in the TE treatment, and the atractylodin concentration of *A. lancea* was lowest in the CO treatment and the monoculture. In 2016, the hinesol and β-eudesmol concentrations of *A. lancea* were significantly lower in the TE treatment than in any other treatment. There were no other significant differences in concentrations of the four volatile oils among the treatments in 2015 and 2016.

The concentrations of the four volatile oils increased significantly from 2015 to 2016 in a few treatments. The hinesol concentration of *A. lancea* in the monoculture, the β-eudesmol concentration of *A. lancea* in the CO and PH treatments, and the atractylodin concentration of *A. lancea* in the CO treatment all increased significantly from 2015 to 2016. However, the hinesol concentration of *A. lancea* decreased from 2015 to 2016 in the GM treatment.

### Effects of Different Intercropping Treatments on Volatile Oil Content of *A. lancea*

The content indicates the quality of volatile oil from *A. lancea*. Based on the analysis of the average rhizome weight and the concentrations of the four main volatile oils in *A. lancea*, the content of the four volatile oils was calculated for individual plants. In 2015, the atractylon content of *A. lancea* was significantly higher in the CO treatment than in the GM, TE, and PH treatments. The hinesol content of *A. lancea* was significantly higher in the ZM and GM treatments than in other treatments. The β-eudesmol content was significantly higher in the ZM treatment than in the TE and PH treatments, and the atractylodin content was significantly higher in the ZM treatment than in the other treatments. In 2016, the atractylon content was significantly higher in the TE, ZM, and CO treatments than in the other treatments. The hinesol content was significantly higher in the CO treatment than in the monoculture, and the hinesol content was significantly lower in the GM and TE treatments than in the monoculture. The β-eudesmol content was significantly higher in the ZM and CO treatments than in the monoculture, and the β-eudesmol content was significantly lower in the GM and PH treatments than in the monoculture. The atractylodin content was significantly higher in the CO, ZM, and TE treatments than in the monoculture.

**FIGURE 5:**
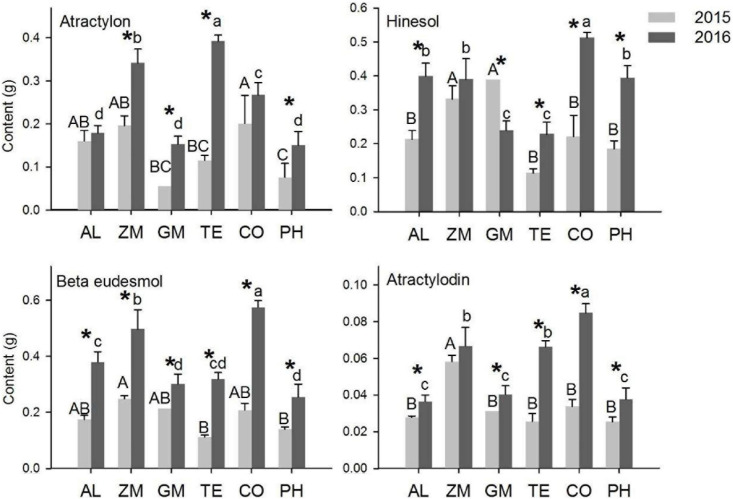
The effect of different intercropping treatments on the contents of four volatile oils in 2015 and 2016. Different capital (2015) and lowercase (2016) letters indicate a significant difference at the 5% significance level, and asterisks indicate significant differences between 2015 and 2016 within individual treatments.

The contents of individual volatile oils increased significantly from 2015 to 2016 in most of the intercropping systems (Figure 5). The atractylon content was significantly higher in 2016 than in 2015 in the TE, ZM, CO, and PH treatments. Likewise, the contents of hinesol (except in the monoculture), β-eudesmol, and atractylodin (except in the ZM treatment) were significantly higher in all treatments in 2016 than in 2015.

### Effects of Different Intercropping Treatments on the Accumulation of Volatile Oils in *A. lancea*

**FIGURE 6:**
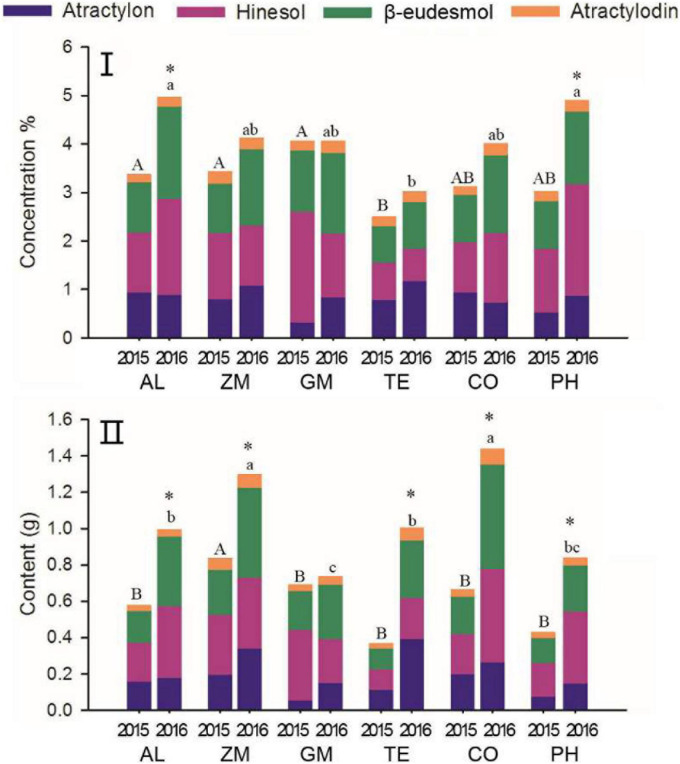
The effect of different intercropping treatments on concentration **(I)** and content **(II)** of total volatile oils in 2015 and 2016. Different capital (2015) and lowercase (2016) letters indicate significant differences at the 5% significance level, and asterisks indicate significant differences between 2015 and 2016 within individual treatments.

The proportion of different volatile oils is an important characteristic of Daodi herbs (Guo et al., 2005). The proportion of individual volatile oil concentrations to the total concentration of all four volatile oils changed markedly from 2015 to 2016 in all treatments (Figure 6, Appendix A.). The total volatile oil concentration increased significantly from 2015 to 2016 in only the AL and PH treatments. The relative concentration of atractylon was higher in 2015 than in 2016 in the AL and CO treatments; the opposite pattern was found in other treatments, particularly for GM, in which the atractylon concentration was significantly higher in 2016. The relative concentration of hinesol was significantly higher in 2015 than in 2016 for the ZM, GM, and TE treatments, and this difference was significant for GM. With the exception of the PH treatment, the relative concentration of β-eudesmol was higher in 2016 than in 2015 for all treatments. The relative concentration of atractylodin did not change from 2015 to 2016 in the GM and TE treatments, whereas its relative concentration was lower in 2016 than in 2015 for the other treatments.

As biomass increased through time, the total volatile oil content increased in all treatments (Figure 6(II)) and increased significantly in the AL, ZM, TE, CO, and PH treatments. Total volatile oil content was highest in the CO treatment, followed by the ZM treatment; it was lowest in the GM treatment. Although the increase in content of individual volatile oils was slight in some treatments, the contents of all four individual volatile oils increased with time in all treatments (with the exception of hinesol in the GM treatment). Contents of individual volatile oils also differed among the treatments. For example, the AL and CO treatments showed clear increases in contents of all four volatile oils, whereas the ZM treatment showed increases primarily in atractylon and β-eudesmol content. The TE treatment showed increases mainly in atractylon and β-eudesmol content, and the PH treatment showed increases mainly in atractylon, β-eudesmol and hinesol content.

**FIGURE 7:**
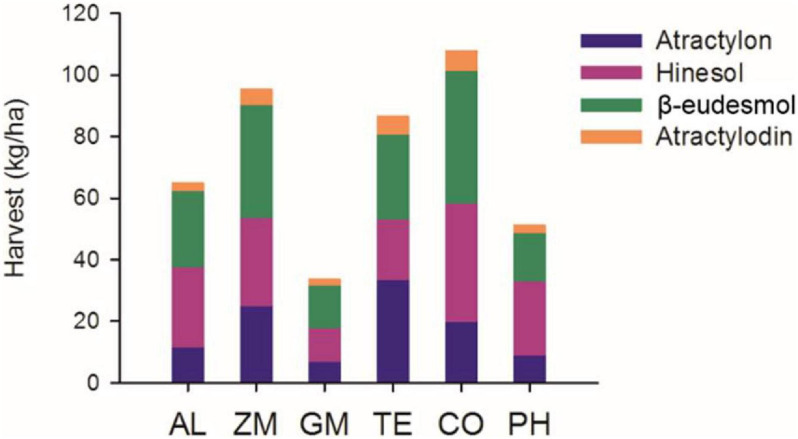
The harvest of total volatile oils from different intercropping treatments in 2016.

Based on plant survival, rhizome weight, and the number of plants per hectare, we calculated the total harvest of the four volatile oils in 2016 (Figure 7). The total volatile oil harvest was higher in the CO, ZM, and TE treatments than in the AL monoculture, whereas that of other treatments was lower than in the AL monoculture.

## Discussion

### Improved Biomass of *A. lancea* Under Intercropping

Complementary patterns of root distribution and plant phenology are important mechanisms by which intercropping improves yield (Keating and Carberry, 1993; Martin-Guay et al., 2018). Intercropping a deeply rooted plant with a more shallowly rooted plant efficiently utilizes belowground space and reduces root competition (Li et al., 2001; Chapagain et al., 2018; Zhang et al., 2020). Phenological complementation of different species can also reduce nutrient competition and increase resource use efficiency. In this study, ZM, TE, and CO treatments markedly improved biomass and the accumulation of four volatile oils in *A. lancea*. In the ZM treatment, *A. lancea* has a shallow root system, whereas ZM has a deep root system. In addition, the phenological phases of ZM, TE, and CO differ from those of *A. lancea*, thereby potentially providing suitable environmental conditions compared with the monoculture.

### Effect of Root Exudates on Survival of *A. lancea*

Root exudates play an important role in plant health (Bais et al., 2006; Sasse et al., 2018; Olanrewaju et al., 2019). In the second year of intercropping, plant survival was higher in the ZM, TE, and CO treatments than in the other treatments (Figure 2), perhaps related to the growth-promoting effect of their root exudates. For example, maize root exudates can enrich plant growth-promoting rhizobacteria and enhance the metabolic capacity of soil bacteria (Baudoin et al., 2002; Benizri et al., 2002; Mendes et al., 2013; Vejan et al., 2016). Moreover, studies have confirmed that the root exudates of CO can inhibit the occurrence of pests and diseases, and it is widely used for this purpose in the field (Ploeg, 2000). *Tagetes erecta* L. has a similar inhibitory effect on root-knot nematodes (Steiner, 1941) and can effectively reduce the density of harmful nematodes (Akhtar and Mashkoor Alam, 1992; Reynolds et al., 2000).

### Effects of Shading on the Accumulation of Four Volatile Oils in *A. lancea*

The accumulation of secondary metabolites is an important index for the evaluation of medicinal materials (Zofou et al., 2013; Song et al., 2014). Secondary metabolites are small molecular organic substances that can assist plants in adapting to the external environment (Kong et al., 2016). In our previous experiments, we found that shading increased the biomass and the content of four volatile oils in *A. lancea* in the short term (Li, 2018). We speculate that the shading effect of the maize plant is responsible for the increased volatile oil content of *A. lancea*. Maize was the tallest plant in this study, and the first year’s results showed that the atractylodin content was significantly higher in the ZM treatment than in the monoculture. Likewise, in 2016, the total volatile oil harvest of *A. lancea* was significantly higher in the ZM treatment than in the monoculture and was the second highest among all the treatments.

## Conclusion

Five plant species were chosen as intercropping partners for *A. lancea*, and the growth traits, survival, and volatile oil production of *A. lancea* were analyzed to evaluate each intercropping combination. Compared with the monoculture, intercropping with ZM, CO, and TE significantly increased the survival and rhizome weight of *A. lancea*. Two years after planting, *A. lancea* intercropped with ZM, TE, and CO showed a great advantage in total volatile oil harvest. The underlying mechanisms of plant interaction in these systems remain to be explored in the future.

## Data Availability Statement

The original contributions presented in the study are included in the article/supplementary material, further inquiries can be directed to the corresponding authors.

## Author Contributions

ZP: writing – original draft and data curation. YZ: investigation, methodology, project administration, and funding acquisition. BY, YX, XG, ZZ, XlC, XpC, and XW: data curation. TW, SW, and CK: data curation and methodology. LH: conceptualization and supervision. LG: investigation, conceptualization, supervision, project administration, and funding acquisition. KS: sample collection, conceptualization, methodology, data curation, and writing – review and editing. All authors contributed to the article and approved the submitted version.

## Conflict of Interest

The authors declare that the research was conducted in the absence of any commercial or financial relationships that could be construed as a potential conflict of interest.

## Appendix A

The relative proportion of individual volatile oils to all four volatile oils in *A. lancea* in 2015 and 2016.

**Appendix A:**

**Treatment**	**Atractylon (%)**		**Hinesol (%)**		**β-eudesmol (%)**		**Atractylodin (%)**	
	**2015**	**2016**	**2015**	**2016**	**2015**	**2016**	**2015**	**2016**
AL	27.87	18.02	36.99	40.11	30.32	38.19	4.83	3.67
ZM	23.54	26.39	39.71	30.07	29.76	38.40	6.98	5.15
GM	8.11	20.89	56.36	32.58	30.99	41.03	4.54	5.50
TE	31.59	39.05	30.92	22.66	30.53	31.71	6.96	6.58
CO	30.32	18.58	33.23	35.61	31.35	39.91	5.10	5.89
PH	17.90	18.01	43.33	47.02	32.81	30.47	5.96	4.50
